# Association between ESR1 rs2234693 single nucleotide polymorphism and uterine fibroids in Taiwanese premenopausal and postmenopausal women

**DOI:** 10.1186/s41043-023-00357-7

**Published:** 2023-03-08

**Authors:** Yeu-Sheng Tyan, Chao-Yu Shen, Disline Manli Tantoh, Shu-Yi Hsu, Ying-Hsiang Chou, Oswald Ndi Nfor, Yung-Po Liaw

**Affiliations:** 1 Department of Medical Imaging, Chung Shan Medical University Hospital, Taichung City, 40201 Taiwan; 2 School of Medicine, Chung Shan Medical University, Taichung City, 40201 Taiwan; 3 School of Medical Imaging and Radiological Sciences, Chung Shan Medical University, Taichung City, 40201 Taiwan; 4 Medical Imaging and Big Data Center, Chung Shan Medical University Hospital, Taichung City, 40201 Taiwan; 5 School of Medical Informatics, Chung Shan Medical University, Taichung City, 40201 Taiwan; 6 Department of Public Health and Institute of Public Health, Chung Shan Medical University, No. 110, Sec. 1 Jianguo N. Rd., Taichung City, 40201 Taiwan; 7 Department of Radiation Oncology, Chung Shan Medical University Hospital, Taichung, 40201 Taiwan; 8 Institute of Medicine, Chung Shan Medical University, Taichung City, 40201 Taiwan

**Keywords:** Women’s health issues, Uterine fibroids, ESR1, rs2234693, Menopausal status

## Abstract

**Background:**

Uterine fibroids (UFs) are uterine smooth muscle neoplasms that affect women, especially during the reproductive stage. Both genetic and lifestyle factors affect the onset of the disease. We examined the association between the estrogen receptor 1 (ESR1) rs2234693 variant (whose genotypes are TT, TC, and CC) and UFs in Taiwanese premenopausal and postmenopausal women.

**Methods:**

We linked individual-level data of 3588 participants from the Taiwan Biobank to the National Health Insurance Research Database at the Health and Welfare Data Science Center. The association of the ESR1 rs2234693 variant and other variables with UFs was determined by multiple logistic regression, and the results were presented as odds ratios and 95% confidence intervals (CIs).

**Results:**

The 3588 participants comprised 622 cases and 2966 controls. In all the participants, the ESR1 rs2234693 TC and CC genotypes compared to the reference genotype (TT) were associated with a lower risk of UFs. However, the results were significant only for the CC genotype (OR; 95% CI = 0.70; 0.52–0.93). Noteworthy, the association of TC and CC with UFs was dose-dependent (*p-trend* = 0.012). Based on menopausal status, both TC and CC were significantly and dose-dependently associated with a lower risk of UFs in premenopausal women (OR; 95% CI = 0.76; 0.59–0.98 for TC and 0.64; 0.43–0.95 for CC: *p-trend* = 0.010).

**Conclusion:**

The TC and CC genotypes of the ESR1 rs2234693 variant may reduce susceptibility to UFs, especially in premenopausal women.

## Background

Uterine leiomyomas, commonly known as uterine fibroids (UFs) or simply fibroids, are non-cancerous smooth muscle uterine tumors that affect women [1–3]. They are the most common gynecologic tumors and affect almost 50–80% of women of childbearing age [4–8]. They have substantial obstetrical consequences that adversely affect women’s well-being [9–11]. Some of the clinical complications of UFs include infertility, severe pelvic pain, miscarriage, abortion, and anemia due to excessive menstrual bleeding [12–15]. Even though UFs are associated with these remarkable complications, their etiology is intricate and remains poorly elucidated [4, 7, 16]. Fibroids stem from both genetic and non-genetic sources including, genetic polymorphisms, menopausal status, alcohol consumption, age, education, cigarette smoking, physical activity, body mass index (BMI), parity, diet, caffeine intake, hypertension, age at menarche, and hormones [1, 5, 7, 16–23].

Hormonal factors play a critical role in the development and progress of UFs [24, 25]. For instance, UFs are estrogen-dependent: as estrogen levels increase, the risk of the disease increases [1, 26, 27]. The rare occurrence of UFs before menarche and a low incidence after menopause support this hypothesis [25]. Moreover, fibroids contain more estrogen receptors than normal adjacent myometrial tissues [25, 28]: The UF-driving nature of estrogen paves a way for uniquely managing the disease by targeting potential estrogen receptors [25]. This is because estrogen affects some of the pathological pathways involved in the pathogenesis of UFs by binding to such receptors [29]. Therefore, fluctuations in the levels of both estrogen and estrogen receptors are implicated in the pathobiology of UFs [25]. Estrogen receptor alpha (ESRα), which is encoded by the estrogen receptor 1 (ESR1) gene [29], is mostly expressed in uterine tissues [25, 30]. This receptor is substantial in the functioning of estrogen in premenopausal and menopausal women [31, 32]. Moreover, it is believed to be among the key elements underlying the pathophysiology of gynecological disorders such as UFs and endometriosis [27, 33–35].

Genomic variations are among the prospective processes underlying the onset of UFs and could contribute to the development of unique therapeutic approaches [25]. A single nucleotide polymorphism (SNP) is a genetic variation at a specific position in a DNA sequence, where a single nucleotide (A, T, C, or G) is substituted by another in at least 1% of the population [36]. Such a variation could affect the structure and function of DNA, thereby conferring disease resistance or susceptibility [37, 38]. Single nucleotide polymorphisms (SNPs) play a key role in the effective prevention of diseases because they serve as disease markers that assist in the early identification of at-risk individuals [39]. For instance, ESR1 PvuII (rs2234693), a genetic variation caused by a nucleotide change from T to C (T > C) is the most studied variant of the ESR1 gene [22, 40–42]. It has been associated with an increased risk of UFs in Taiwanese [35], Black, and White American women [33], but not in German [43], Polish [42], Italian [44, 45], Hispanic [33], and Iranian women [46]. Given this controversial relationship between the variant and UFs, further epidemiological research is needed. Furthermore, studies with large sample sizes have been recommended to clarify the relationship between rs2234693 and UFs [42]. Therefore, we carried out this study to determine the association between the ESR1 rs2234693 SNP and UFs in Taiwanese premenopausal and postmenopausal women.

## Materials and methods

### Ethical compliance

Ethical approval for this work was granted by the Institutional Review Board of the Chung Shan Medical University Hospital (CS2-20006). All participants signed an informed consent form before enrolling in the TWB project.

### Participants and datasets

We enrolled 3994 participants (1994 premenopausal and 2000 postmenopausal women with complete data) who were recruited into the Taiwan Biobank (TWB) project between 2008 and 2015. The TWB project was created to collect and integrate genetic and non-genetic data of over 200,000 Taiwanese adults aged between 30 and 70 years, with no cancer diagnosis to undertake large-scale cohort and case–control studies [47]. At the time of the current study, the TWB database contained basic demographic information (e.g., age, sex, and education), personal lifestyle habits (e.g., exercise, smoking, alcohol, tea, and coffee consumption), and genetic data (e.g., single nucleotide polymorphisms). However, information on uterine fibroids was not available in the database. Nonetheless, the National Health Insurance Research Database (NHIRD) contained data on uterine fibroids. To determine the risk of UFs, we used the participants’ identification numbers and linked the TWB database (2008–2015) to the NHIRD (1998–2015) at the Health and Welfare Data Center (HWDC). The HWDC is a data repository site established by the Ministry of Health and Welfare (MOHW). This center allows the linking and management of several databases under strict supervision to ensure data privacy and security [48]. We excluded 406 menopausal women whose menopause was not attained naturally. Finally, our study included 3588 participants, comprising 1594 premenopausal and 1994 postmenopausal women.

### Identification and definition of variables

We chose rs2234693 because it is associated with female reproduction and is one of the most commonly assessed ESR1 SNPs [40, 49]. Genotyping experiments were performed by the National Center for Genome Medicine in Academia Sinica using a custom Affymetrix Axiom Genome-Wide Array Plate (Affymetrix Inc. Santa Clara, CA, USA) called TWB chip. During genotyping, SNPs that failed quality control: had a minor allele frequency (MAF) < 0.05, a call rate < 95%, and deviated from the Hardy–Weinberg equilibrium (HWE)—*p* value < 1.0 × 10^−3^—were excluded. The MAF, call rate, and HWE p value for rs2234693 were 0.378887, 99.79%, and 0.2174, respectively.

Diseases in the NHIRD were identified using the International Classification of Diseases, Ninth Revision, Clinical Modification (ICD-9-CM) codes and a single admission or two outpatient visits. The ICD-9-CM codes were 218.0, 218.1, 218.2, and 218.9 for UFs [50], 401–405, A260, and A269 for hypertension, and then 250 and A181 for diabetes mellitus.

Lifestyle habits, menopausal status, age, educational level, age at menarche, family history of UFs, miscarriage/abortion, and parity were self-reported. The postmenopausal subjects were women who reported an absence of menstrual flow (not due to hysterectomy or any medical condition/treatment) for at least twelve consecutive months, while the premenopausal subjects included women who were still experiencing monthly menstrual bleeding at the time of the interview. An elaborate description of the other variables has been provided elsewhere [51, 52]. In summary, we defined alcohol consumption as a weekly intake of at least 150 ml of alcohol over 6 months; smoking as regular use of cigarettes over 6 months; exercise as engaging in at least 30 min of exercise (excluding manual work) ≥ 3 times per week; coffee consumption as drinking coffee at least three times per week; and tea consumption as drinking tea at least once per day. We defined a vegetarian as someone who maintained a vegetarian lifestyle for at least 6 months prior to data collection; use of hormones as regular use of western hormonal medicine for more than 6 months; use of herbal medicine as the use of herbs (for gynecological conditions such as menstruation and menopause) for 3 months; and second-hand smoke exposure as being exposed to tobacco smoke for at least 5 min per hour. BMI was calculated as weight (kg) divided by height squared (m^2^).

### Statistical analyses

The *t* test and chi-squared test were used to evaluate the differences between continuous and non-continuous (categorical) variables, respectively. We determined the association between ESR1 rs2234693 and uterine fibroid using the multiple logistic regression analysis. Adjustments were made for menopausal status, alcohol consumption, age, education, cigarette smoking, exercise, second-hand smoke exposure, hypertension, tea/coffee consumption, vegetarian diet, age at menarche, hormone use, herbal medicine use, family history of uterine fibroid, miscarriage/abortion, BMI, and parity. SAS (version 9.4) was used to perform statistical analyses, while PLINK (version 1.09) was used for SNP quality control [53].

## Results

The participants comprised 622 cases of uterine fibroids and 2966 controls (Table 1). The difference in the rs2234693 genotype distribution between the cases and controls was significant at borderline (*p* = 0.052). Age, hypertension, diabetes, the use of herbal medicine, family history of uterine fibroids, and miscarriage/abortion were significantly different between the cases and controls (*p* < 0.05).

**Table 1:** Basic characteristics of participants with uterine fibroids (cases) and without uterine fibroids (controls)

Variable	Controls (*n* = 2966)	Cases (*n* = 622)	*p* value
Categorical variables	*n* (%)	*n* (%)	
ESR1 rs2234693 genotype			0.052
TT	1132 (38.17)	263 (42.28)	
TC	1392 (46.93)	286 (45.98)	
CC	442 (14.90)	73 (11.74)	
Menopause			0.678
No	1653 (55.73)	341 (54.82)	
Yes	1313 (44.27)	281 (45.18)	
Alcohol consumption			0.321
No	2845 (95.92)	595 (95.66)	
Yes	121 (4.08)	27 (4.34)	
Age (years)			< 0.001*
30–39	685 (23.10)	63 (10.13)	
40–49	829 (27.95)	210 (33.76)	
50–59	838 (28.25)	254 (40.84)	
60–69	614 (20.70)	95 (15.27)	
Level of education			0.187
Elementary school	289 (9.74)	55 (8.84)	
High school	1473 (49.66)	334 (53.70)	
University and above	1204 (40.59)	233 (37.46)	
Cigarette smoking			0.766
No	2845 (95.92)	595 (95.66)	
Yes	121 (4.08)	27 (4.34)	
Exercise			0.163
No	1721 (58.02)	342 (54.98)	
Yes	1245 (41.98)	280 (45.02)	
Second-hand smoke exposure			0.104
No	2651 (89.38)	542 (87.14)	
Yes	315 (10.62)	80 (12.86)	
Hypertension			0.004*
No	2404 (81.05)	473 (76.05)	
Yes	562 (18.95)	149 (23.95)	
Diabetes			0.414
No	2610 (88.00)	540 (86.82)	
Yes	356 (12.00)	82 (13.18)	
Tea consumption			0.825
No	2059 (69.42)	429 (68.97)	
Yes	907 (30.58)	193 (31.03)	
Coffee consumption			0.307
No	1980 (66.76)	402 (64.93)	
Yes	986 (33.24)	220 (35.37)	
Vegetarian diet			0.361
No	2648 (89.28)	563 (90.51)	
Yes	318 (10.72)	59 (9.49)	
Hormone use			0.069
No	2558 (86.24)	519 (83.44)	
Yes	408 (13.76)	103 (16.56)	
Herbal medicine use			0.006*
No	2679 (90.32)	539 (86.66)	
Yes	287 (9.68)	83 (13.34)	
Family history of uterine fibroids			< 0.001*
No	2546 (85.84)	488 (78.46)	
Yes	420 (14.16)	134 (21.54)	
Miscarriage/abortion			< 0.001*
No	1170 (39.45)	197 (31.67)	
Yes	1796 (60.55)	425 (68.33)	
Age at menarche (years)			0.943
Age at menarche ≤ 12	603 (20.33)	126 (20.26)	
12 < age at menarche ≤ 13	786 (26.50)	172 (27.65)	
13 < age at menarche ≤ 14	897 (30.24)	183 (29.42)	
Age at menarche > 14	680 (22.93)	141 (22.67)	
Continuous variables			
Body mass index (kg/m^2^)	23.48 ± 3.412	23.62 ± 3.215	0.340
Parity	2.32 ± 0.978	2.29 ± 0.888	0.481

*n* sample size, *ESR1* Estrogen receptor 1, *SD* standard deviation

*Denotes statistical significance at *p* < 0.05

Table 2 presents the association between rs2234693 and UFs in all of the 3588 participants. Compared to the TT genotype (reference), the CC genotype was associated with a lower risk of UFs (odds ratio OR = 0.70, 95% confidence interval CI 0.52–0.93), while the TC genotype was not significantly associated with the disorder (OR = 0.87, 95% CI 0.72–1.05). This indicates a 30% lower likelihood of having UFs in the subjects with the CC genotype compared with those with the TT genotype. Of note, the relationship of the genotypes with UFs was dose-dependent (*p-trend* = 0.012). A lower risk of UFs was found in the menopause group (OR = 0.69, 95% CI 0.51–0.92), while a higher risk of the disorder was seen in women who were 40–49 years old (OR = 2.96, 95% CI 2.16–4.06), 50–59 years old (OR = 4.55, 95% CI 3.09–6.69), 60–69 years old (OR = 2.59, 95% CI 1.59–4.21), hypertensive (OR = 1.32, 95% CI 1.04–1.67), using herbal medicine (OR = 1.50, 95% CI 1.14–1.97), having a family history of UFs (OR = 1.60, 95% CI 1.28–2.00), and/or having a history of miscarriage/abortion (OR = 1.24, 95% CI 1.02–1.49).

**Table 2:** Association between ESR1 rs2234693 and uterine fibroids

Variable	OR (95% CI)	*p* value
*ESR1 rs2234693 (ref.: TT)*		
TC	0.87 (0.72–1.05)	0.147
CC	0.70 (0.52–0.93)	0.015*
*p-trend*	0.012*	
*Menopause (ref.: No)*		
Yes	0.69 (0.51–0.92)	0.012*
*Alcohol consumption (ref.: No)*		
Yes	1.25 (0.71–2.21)	0.445
*Age (ref.: 30–39 years)*		
40–49	2.96 (2.16–4.06)	< 0.001*
50–59	4.55 (3.09–6.69)	< 0.001*
60–69	2.59 (1.59–4.21)	< 0.001*
*Level of education (ref.: Elementary school)*		
High school	1.05 (0.75–1.48)	0.780
University and above	1.00 (0.69–1.44)	0.982
*Cigarette smoking (ref.: No)*		
Yes	1.01 (0.64–1.60)	0.9708
*Exercise (ref.: No)*		
Yes	1.02 (0.84–1.23)	0.876
BMI	0.99 (0.97–1.02)	0.659
*Second-hand smoke exposure (ref.: No)*		
Yes	1.25 (0.95–1.64)	0.117
*Diabetes (ref.: No)*		
Yes	1.06 (0.80–1.40)	0.697
*Hypertension (ref.: No)*		
Yes	1.32 (1.04–1.67)	0.021*
*Tea consumption (ref.: No)*		
Yes	1.03 (0.85–1.26)	0.765
*Coffee consumption (ref.: No)*		
Yes	1.05 (0.86–1.27)	0.631
*Vegetarian diet (ref.: No)*		
Yes	0.84 (0.62–1.13)	0.254
*Age at menarche (ref.: age at menarche ≤ 12)*		
12 < age at menarche ≤ 13	0.93 (0.72–1.21)	0.611
13 < age at menarche ≤ 14	0.87 (0.67–1.13)	0.302
Age at menarche > 14	0.88 (0.66–1.17)	0.372
*Hormone use (ref.: No)*		
Yes	1.21 (0.94–1.55)	0.147
*Herbal medicine use (ref.: No)*		
Yes	1.50 (1.14–1.97)	0.004*
*Family history of uterine fibroids (ref.: No)*		
Yes	1.60 (1.28–2.00)	< 0.001*
*Miscarriage/abortion (ref.: No)*		
Yes	1.24 (1.02–1.49)	0.030*
Parity	0.91 (0.82–1.01)	0.066

*ESR1* Estrogen receptor 1, *OR* Odds ratio, *CI* Confidence interval, *ref.* reference

*Denotes statistical significance at *p* < 0.05

Table 3 shows the association between ESR1 rs2234693 and UFs in premenopausal and postmenopausal women. Compared to the reference genotype (TT), both the TC and CC genotypes were significantly and dose-dependently associated with a lower risk of UFs in premenopausal women (OR = 0.76, 95% CI = 0.59–0.98 for TC and OR = 0.64, 95% CI = 0.43–0.95 for CC: *p-trend* = 0.010). However, the TC and CC genotypes were not associated with the occurrence of UFs in postmenopausal women. Age at menarche had an inverse but insignificant relationship with UFs in premenopausal women. This inverse relationship was dose-dependent (*p-trend* = 0.030).

**Table 3:** Association between ESR1 rs2234693 and uterine fibroids stratified by menopausal status

Variable	No menopause		Menopause	
	OR (95% CI)	*p* value	OR (95% CI)	*p* value
*ESR1 rs2234693 (ref.: TT)*				
TC	0.76 (0.59–0.98)	0.0371*	1.06 (0.79–1.40)	0.714
CC	0.64 (0.43–0.95)	0.0263*	0.78 (0.50–1.20)	0.255
*P-trend*	0.010*	0.431		
*Alcohol consumption (ref.: No)*				
Yes	1.29 (0.63–2.64)	0.482	1.21 (0.46–3.17)	0.698
*Age*				
30–39	Ref	–	NA	NA
40–49	2.88 (2.08–3.99)	< 0.001*	Ref	–
50–59	4.14 (2.71–6.31)	< 0.001*	2.18 (0.97–4.94)	0.061
60–69	NA	NA	1.18 (0.51–2.74)	0.693
*Level of education (ref.: Elementary school)*				
High school	0.93 (0.43–2.02)	0.858	1.07 (0.72–1.59)	0.728
University and above	0.82 (0.37–1.80)	0.616	1.20 (0.76–1.88)	0.437
*Cigarette smoking (ref.: No)*				
Yes	0.96 (0.55–1.68)	0.896	1.07 (0.46–2.47)	0.880
*Exercise (ref.: No)*				
Yes	1.07 (0.82–1.40)	0.616	0.93 (0.70–1.22)	0.585
BMI	1.00 (0.96–1.04)	0.994	0.98 (0.93–1.02)	0.313
*Second-hand smoke exposure (ref.: No)*				
Yes	1.23 (0.87–1.74)	0.239	1.30 (0.82–2.06)	0.271
*Diabetes (ref.: No)*				
Yes	1.22 (0.76–1.96)	0.417	0.96 (0.68–1.36)	0.825
*Hypertension (ref.: No)*				
Yes	1.20 (0.80–1.78)	0.375	1.40 (1.04–1.88)	0.025*
*Tea consumption (ref.: No)*				
Yes	0.96 (0.74–1.25)	0.763	1.15 (0.84–1.57)	0.375
*Coffee consumption (ref.: No)*				
Yes	1.16 (0.90–1.50)	0.243	0.90 (0.66–1.22)	0.481
*Vegetarian diet (ref.: No)*				
Yes	1.12 (0.78–1.62)	0.541	0.49 (0.29–0.85)	0.012*
*Age at menarche (ref.: age at menarche* ≤ *12)*				
12 < age at menarche ≤ 13	0.98 (0.71–1.37)	0.911	0.88 (0.57–1.37)	0.583
13 < age at menarche ≤ 14	0.82 (0.58–1.15)	0.239	0.92 (0.61–1.41)	0.712
Age at menarche > 14	0.67 (0.44–1.00)	0.051	1.10 (0.72–1.68)	0.656
*P-trend*	0.030*	NA		
*Hormone use (ref.: No)*				
Yes	1.19 (0.79–1.79)	0.408	1.23 (0.89–1.69)	0.217
*Herbal medicine use (ref.: No)*				
Yes	1.68 (1.21–2.36)	0.002*	1.22 (0.73–2.03)	0.454
*Family history of uterine fibroids (ref.: No)*				
Yes	1.88 (1.41–2.51)	< 0.001*	1.24 (0.86–1.80)	0.258
*Miscarriage/abortion (ref.: No)*				
Yes	1.40 (1.08–1.82)	0.012*	1.06 (0.80–1.40)	0.704
Parity	0.95 (0.82–1.10)	0.488	0.87 (0.75–1.02)	0.082

*ESR1* Estrogen receptor 1, *OR* Odds ratio, *CI* Confidence interval, *ref.* Reference, *NA* Not applicable

*Denotes statistical significance at *p* < 0.05

Table 4 shows the risk of UF based on the combination of the ESR1 rs2234693 genotypes and menopausal status. Compared to the reference group (premenopausal women with the TT genotype), the risk of UF was significantly lower in the other groups including, premenopausal women with TC (OR = 0.75, 95% CI 0.58–0.97), premenopausal women with CC (OR = 0.66, 95% CI 0.45–0.97), postmenopausal women with TT (OR = 0.58, 95% CI 0.41–0.84), postmenopausal women with TC (OR = 0.60, 95% CI 0.42–0.85), and postmenopausal women with CC (OR = 0.43, 95% CI 0.27–0.70).

**Table 4:** Risk of uterine fibroids based on ESR1 rs2234693 genotypes and menopausal status

Variable	*n*	OR (95% CI)	*p* value
ESR1 rs2234693 genotypes and menopausal status (ref.: TT, no menopause)	775		
TC, no menopause	927	0.75 (0.58–0.97)	0.030*
CC, no menopause	292	0.66 (0.45–0.97)	0.034*
TT, menopause	620	0.58 (0.41–0.84)	0.004*
TC, menopause	751	0.60 (0.42–0.85)	0.004*
CC, menopause	223	0.43 (0.27–0.70)	0.001*

Adjusted for age, level of education, cigarette smoking, exercise, BMI, second-hand smoke exposure, diabetes, hypertension, tea/coffee consumption, vegetarian diet, age at menarche, hormone/herbal medicine use, family history of uterine fibroids, miscarriage/abortion, and parity

*ESR1* Estrogen receptor 1, *OR* Odds ratio, *CI* Confidence interval, *ref*. Reference

*Denotes statistical significance at *p* < 0.05

## Discussion

In the current study, the risk of UFs was significantly lower in postmenopausal Taiwanese women compared to their premenopausal counterparts. Both the TC and CC genotypes of the ESR1 rs2234693 SNP were significantly associated with a lower risk of UFs among premenopausal women, implying that the ESR1 rs2234693 variant might protect against UFs. Each population has its unique genetic characteristics which could affect its susceptibility to diseases. As such, it is important to determine the effect of genetic variants on health outcomes in specific populations because findings from one ethnic population might not be directly applicable to another [54]. Since our study subjects were exclusively Taiwanese, our findings add to the knowledge regarding the potential genetic determinants of uterine fibroid (a non-communicable disease) in the Taiwanese population.

Most UF-related pathways are complex [55]. The role of the ESR1 gene in the pathogenesis of UFs and other gynecologic diseases has been reported [27, 33–35, 56]. Regarding UF pathobiology, the role of ESR1 is attributed, in part to rs2234693 [33, 35, 42]. This genetic variant alters the binding of transcription factors and affects alternative splicing of ESR1, thereby influencing its expression and functionality [29, 34, 57, 58]. Higher ESR1 expression resulting from rs2234693 C allele-induced transcription could enhance oestradiol-ESR1 binding, subsequently leading to a stronger response to estradiol in CC homozygous women compared to T allele carriers [32]. Menopausal women carrying the rs2234693 T allele have been found to have the lowest levels of estradiol while those with the CC genotypes have the highest levels [59]. Moreover, in a previous study, low levels of estrogen coupled with the T allele were associated with lower ESR1 expression [57]. In the present study, rs2234693 CC homozygosity was inversely associated with UFs. However, the association was significant only in premenopausal women. The ineffectiveness of the CC genotype in postmenopausal women could be due to estrogen deficiency and probably lower expressions of the receptor [31]. A lower risk of UFs in postmenopausal women compared to premenopausal women has been reported [60]. It is worth noting, however, that the association between ESR1 rs2234693 and UFs remains controversial [40]. In the current study, we observed an inverse association of rs2234693 TC and CC with UFs. So far, we are aware of only one study that explored the relationship between both variables in Taiwanese [35]. In their study, Hsieh and colleagues included 106 cases and 110 controls and found a moderate correlation between the rs2234693 C-related genotype and susceptibility to UFs [35]. This polymorphism was also associated with an increased risk of UFs in Indian [34], as well as black and white American women [33]. In a meta-analysis of 26,428 cases of UFs and 43,381 controls, the T allele of rs2234693 was significantly associated with a lower risk of UFs [40]. In contrast, no significant relationship existed between the polymorphism and UFs in Polish [42], German [43], Iranian [46], and Italian women [44, 45].

In the current study, age was positively associated with UFs. That is, older age was significantly associated with a higher risk of UFs, confirming the evidence that the risk of UFs increases with age, especially during the reproductive stage [1, 19, 21, 61]. However, age at menarche among premenopausal women was inversely associated with UFs in a dose-dependent manner. That is, increasing age at menarche was significantly associated with a decreasing risk of UFs. The inverse relationship between age at menarche and UFs has been previously reported [1, 18, 26, 62]. The present study suggests that vegetarian diet could be associated with a lower risk of UFs among postmenopausal women; this agrees with previous studies [63, 64]. Similar to the current findings, UFs have been positively associated with hypertension [65–67], family history [21, 42, 68–70], abortion, and miscarriage [23, 71]. More is yet to be explored regarding the effect of herbal medicine on the onset of UFs. So far, some studies have reported a lower risk of UFs among people taking Chinese traditional medicine [72, 73]. According to Li and colleagues [74], several herbal medicines and natural products are used as alternative therapies for UFs due to their antiinflammatory, antiproliferative, and antiangiogenic activities. In the current study, however, the use of herbal medicine was associated with a higher risk of UFs.

The strength of the current study is that it is the first to link two important research databases in Taiwan (TWB and NHIRD) to ascertain participants’ genetic and non-genetic information and determine the risk of UFs. However, the limitation is that the Taiwan Biobank project enrolled only Taiwanese adults aged 30–70 years. As such, this study was unable to determine the risk of UFs in women aged below 30 and above 70 years. In this sense, our results may not be generalized to all Taiwanese premenopausal and postmenopausal women. Furthermore, this study only suggests the possible association between ESR1 rs2234693 and uterine fibroids and cannot establish causality due to its design.

## Conclusions

Both the ESR1 rs2234693 TC and CC genotypes may decrease the risk of UFs, particularly in premenopausal women. However, older age, early menarche, family history of UFs, and miscarriage/abortion may increase the risk. The clinical implication of these results is that the ESR1 rs2234693 variant might protect against UFs. This study contributes to the knowledge about the role of genetic factors in the pathogenesis of UFs. We hope that our results will serve as a reference for future studies evaluating the genetic factors involved in the pathogenesis of fibroids.

## Data Availability

The data that support the findings of this study are available from Taiwan Biobank but restrictions apply to the availability of these data, which were used under license for the current study, and so are not publicly available. Data are however available from the authors upon reasonable request and with permission of Taiwan Biobank.

## Abbreviations

BMI: Body mass index
CI: Confidence interval
ESR1: Estrogen receptor 1
ESRα: Estrogen receptor alpha
ICD-9-CM: International classification of diseases, ninth revision, clinical modification
HWDC: Health and Welfare Data Science Center
MAF: Minor allele frequency
MOHW: Ministry of Health and Welfare
MOST: Ministry of Science and Technology
NHIRD: National Health Insurance Research Database
OR: Odds ratio
SD: Standard deviation
SNP: Single nucleotide polymorphism
TWB: Taiwan biobank
UFs(s): Uterine fibroid(s)
